# Menstrual changes after COVID-19 vaccination and/or SARS-CoV-2 infection and their demographic, mood, and lifestyle determinants in Arab women of childbearing age, 2021

**DOI:** 10.3389/frph.2022.927211

**Published:** 2022-07-22

**Authors:** Ahmed A. Amer, Samar A. Amer, Khaled Masoud Alrufaidi, Eman Elsayed Abd-Elatif, Bana Zuhair Alafandi, Dalia Abdelmonaim Yousif, Nadia Toukah Armi, Atheer Abdulaziz Alkhalaf, Jaffer Shah, Mohamed Sh Ramadan

**Affiliations:** ^1^Department of Obstetrics and Gynecology, Faculty of Medicine, Zagazig University, Zagazig, Egypt; ^2^Department of Public Health and Community Medicine, Faculty of Medicine, Zagazig University, Zagazig, Egypt; ^3^Royal College of General Practioners, London, United Kingdom; ^4^Department of Epidemiology, Faculty of Medicine, King Saud University, Riyadh, Saudi Arabia; ^5^Department of Public Health and Community Medicine, Faculty of Medicine, Mansoura University, Mansoura, Egypt; ^6^Faculty of Medicine, University of Aleppo, Aleppo, Syria; ^7^Faculty of Medicine, Red Sea University, Port Sudan, Sudan; ^8^Faculty of Medicine, Arab Medical University, Benghazi, Libya; ^9^Oral and Dental Medicine and Surgery, Riyadh Elm University, Riyadh, Saudi Arabia; ^10^New York State Department of Health, New York, NY, United States; ^11^Department of Obstetrics and Gynecology, Faculty of Medicine, Zagazig University, Zagazig, Egypt

**Keywords:** menstrual changes, mood, SARS-CoV-2 infection, COVID-19 vaccine, lifestyle, childbearing period

## Abstract

**Background:**

By September 2, 2021, over 30,000 COVID-19-vaccinated females had reported menstrual changes to the MHRA's Yellow Card surveillance system. As a result, the National Institutes of Health (NIH) is urging researchers to investigate the COVID-19 vaccine's effects on menstruation. Therefore, this study was conducted to explore the menstrual changes after COVID-19 vaccination and/or SARS-CoV-2 infection and their interrelations with demographic, mood, and lifestyle factors in Arab women of childbearing age (CBA).

**Methodology:**

A cross-sectional study was conducted during October 2021 using an Arabic validated and self-administrated questionnaire. In total, 1,254 Women of CBA in the Arabic Population (15–50 y) with regular menstrual cycles were randomly selected from five countries (Saudi Arabia, Egypt, Syria, Libya, and Sudan).

**Results:**

The mean (SD) age of the 1,254 studied females was 29.6 (8.5) years old. In total, 634 (50%) were married, 1,104 (88.0%) had a University education or above, 1,064 (84.4%) lived in urban areas, and 573 (45.7%) had normal body weight. Moreover, 524 (41.8%) were COVID-19 cases and 98 women (18.7%) reported menstrual changes (MCs). The 1,044 (83.5%) vaccinated females reported 418 (38.5%) MCs after being vaccinated, and these MCs resolved in 194 women (55.1%) after more than 9 months. Statistically significant relationships were observed between the reported MCs and the following variables: age, marital status, level of education, nationality, residence, and BMI. MCs were reported at 293(80.6) after the 2nd dose, and were mainly reported after 482 (46.1) Pfizer, 254 (24.3) Astrazenica, and 92 (8.8) Senopharm.

**Conclusion:**

MCs among women of CBA after COVID-19 infection and vaccination are prevalent and complex problems, and had many determinates.

## Key message

Cross sectional study targeted 1,254 women of childbearing age to explore the menstrual changes after COVID-19 vaccination and/or SARS-CoV-2 infection and their interrelations with the demographic, mood, and lifestyle factors in Arab women of childbearing age (CBP). Menstrual cycle changes after COVID-19 infection and vaccination are prevalent and complex problem.

## Introduction

Globally, there are 233,479,934 confirmed coronavirus disease 2019 (COVID-19) cases with 4,786,203 deaths and 6,314,235,621 vaccinated cases according to the WHO dashboard on 1 October 2021^1^. The clinical manifestations of COVID-19 disease are heterogeneous (1). The impacts of the COVID-19 pandemic are not gender-neutral.

During the COVID-19 pandemic, over 800 million women undergo menstrual cycles and need safe and hygienic menstrual products. One out of five female athletes reported menstrual cycle changes after the pandemic onset (2). Reports on its impact on menstruation are lacking, except for a very recent study that reported that the menstrual disorders increased during the COVID-19 pandemic but not vaginal infection (3) and found an association between the pandemic-induced depression, anxiety, and perceived stress and the increased prevalence of menstrual irregularity (4).

The most promising intervention to control this pandemic is vaccination. Several vaccines have become available by the end of 2020 in many parts of the world, with many more under trial. Over 40 vaccines in human trials and over 150 vaccines in preclinical were adequately assessed and regularly updated by the World Health Organization (WHO) (5). Although the UK's Medicines and Healthcare Products Regulatory Agency (MHRA) did not list menstrual changes (MCs) or unexpected vaginal bleeding as side effects of the COVID-19 vaccine, the working physicians in reproductive health are increasingly reporting these events after both mRNA and adenovirus-vectored COVID-19 vaccines (6).

MCs are likely to be due to the immune stimulation by the immune cells biologically mediated effect in the uterus lining, which are involved in the cyclical build-up and breakdown of this tissue (7), or immunological influences on the menstrual cycle hormones (9) rather than the vaccine component (6). Although MCs after vaccination are short-lived and do not adversely affect fertility (as there is no sufficient evidence of their impact on fertility) (8), they may adversely affect the success of the vaccination programmer. Vaccination hesitancy among young women is driven by the false claims that vaccines could harm their future pregnancy (9).

By 2 September 2021, more than 30,000 COVID-19-vaccinated females reported MCs through MHRA's Yellow Card surveillance system (6). The Yellow Card evaluation reports do not support a relationship between COVID-19 vaccines and MCs because of both the relatively low number of vaccinated women and the general prevalence of menstrual disorders (10).

Therefore, clear and trusted information is required and essential to build trust regarding vaccine safety and to predict MCs to either achieve or avoid pregnancy. The Royal College of Obstetricians and Gynecologists and the MHRA recommend that any females reporting persisting MCs or new vaginal bleeding after vaccination be managed using the usual clinical guidelines for these conditions (11).

Unfortunately, questions about menstruation were excluded from most large-scale COVID-19 studies (including vaccine trials), so it is currently unknown how many women have experienced menstrual cycle changes, how long these changes persisted, and the extent of their impact. However, a few scientific studies of variable quality have reported on menstrual cycle features during the pandemic, but it is still unclear whether the observed changes are due to the COVID-19 illness or other pandemic-related factors such as increased psychological stress and changes in health behaviors (12).

Recently, Eunice Kennedy Shriver National Institute of Child Health and Human Development (NICHD) released a notice for researchers to compare the menstruation experiences of vaccinated and unvaccinated people and how the vaccine affects menstruation (13). Moreover, WHO encourages and calls for investments in quality and gender-sensitive research on the adverse health, social, and economic impacts of COVID-19 (14). We are still awaiting definitive evidence, and further researches are required to explore and help understand the possible mechanisms. Therefore, this study was conducted to explore the menstrual changes after COVID-19 vaccination and/or SARS-CoV-2 infection and their interrelations with the demographic, mood, and lifestyle factors in Arab women of childbearing age (CBA).

## Methods

### Participants and study design

**Figure 1:**
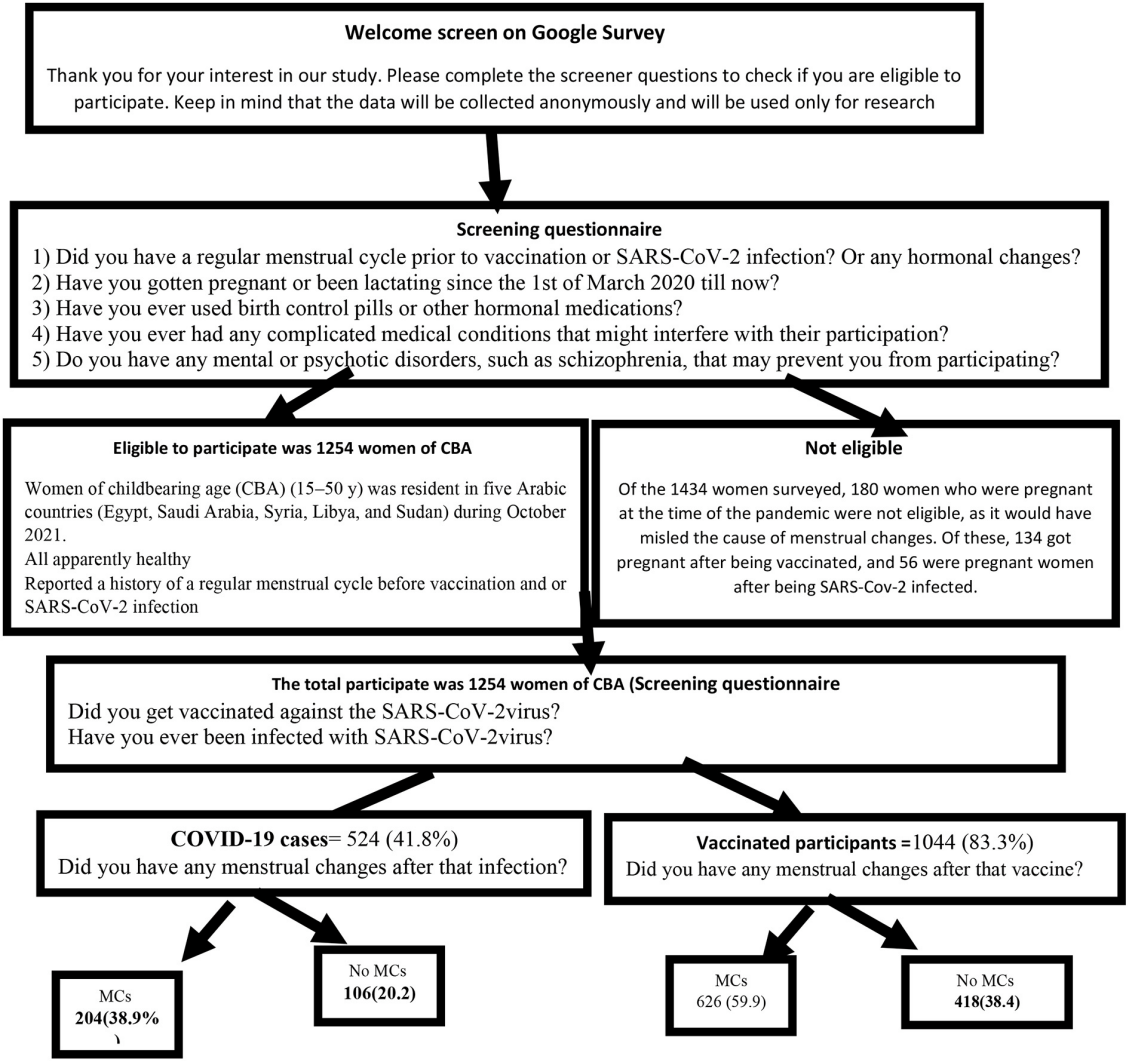
Survey recruitment flow diagram.

A cross-sectional retrospective survey of 1,434 women of childbearing age (CBA) (15–50 y) was conducted in five Arabic countries (Egypt, Saudi Arabia, Syria, Libya, and Sudan) during October 2021. The selection criteria were as follows: women participating in this study were all apparently healthy and reported a history of a regular menstrual cycle before vaccination and or SARS-CoV-2 infection. Illiterate, internet non-users, pregnant women using contraceptive pills, lactating women, women with other hormonal changes or medications, and those with complicated medical, mental, or psychotic disorders, such as schizophrenia, that may interfere with their participation were excluded as in Figure 1.

### Sample size, and sampling techniques

The sample size was estimated according to the following equation: *n* = Z^2^ P (1 – P)/d2. Due to limited data about the prevalence of MCs after COVID-19 vaccinations, based on a previous study that reported that nearly 20% of females infected with COVID-19 had decreased menstrual volume or prolongation of the menstrual cycle (13), at a 95% confidence level and 80% power of the study, the calculated sample size is shown in Supplementary Table 1. In terms of the probable geographical variations per infection rates among women, the data available are only from a few countries and are skewed (15). Therefore, we use the reference population of^2^ (16).

The Women of CBA was recited using a multistage sampling method. We randomly selected five Arabic countries from the Middle East; we chose three governorates at random from each country; and of those three governorates from each country, we chose one urban and one rural area; then, the official websites for the areas in each governorate were used to collect community-based samples. The number of women in CBA and the number of COVID-19 cases or vaccinated people who met all of the selection criteria were used to figure out how many samples were needed from each region.

Participants completed and submitted the questionnaire after giving approval for participation in the study (informed consent). The questionnaire was distributed among the targeted countries and the randomly selected regions and areas, and covered the most common and official media social platforms of these areas (Facebook, Twitter, and WhatsApp groups). Reminder messages and follow-up were used to increase the response rate. Until the required sample is completed.

### Data collection tool

The questionnaire was created and adopted using data from previous studies (11–13). It was developed in English and then translated into Arabic. A bilingual panel including two healthcare experts and one externally qualified medical translator translated the English version of the questionnaire into Arabic. Two English-speaking translators completed a back translation, and the original panel was consulted if there were any concerns.

The data was collected through an Arabic-well-structured, online self-administered questionnaire. We evaluated the questionnaire's reliability and validity to ensure that six different countries interpreted the questions in the same way. Six obstetric and gynecologists, one from each country, validated the questionnaire, which was then assessed for clarity and comprehension in a pilot study that involved 15 CBA women from each country. Their findings were not included in our study. Finally, for the questions about the MCs and how the COVID-19 pandemic affected the participants' lives, we computed a Cronbach's alpha of 0.78. In a number of original studies (17, 18), the tools used to measure depression and anxiety levels had been shown to be accurate.

It was composed of the following sections:

1) Information about the study and solicited informed consent electronically.
2) Sociodemographic and health-related factors: age, sex, residence, educational level, occupation, marital status, weight, and height.
3) Menstrual history, especially for contraceptive pill use and the hormone therapy.
4) History of SARS-CoV-2 infection and /or vaccination (type, doses, and timing).
5) Post COVID-19 infection and /or vaccination menstrual cycle changes.

- Changes in the amount, and duration of menstrual cycles. Amenorrhea, is defined as the absence of three consecutive menstrual cycles during the reproductive years of a woman's life after excluding pregnancy (either by consultation, or pregnancy test) (19).
- Changes in menstrual related symptoms, such as pelvic and abdominal pain, back pain, breast pain, mood swings, headache, fatigue, ovulatory pain, and dyspareunia.

6) Assessment of the level of depression and anxiety:

- Depression Patient Health Questionnaire-2 (PHQ-2) was used to screen for depression in the “first-step” approach and inquiries about the frequency of depressed mood and anhedonia over the past 2 weeks with a Likert scale (0–3) for each criterion. The PHQ-2 score ranges from 0 to 6. It includes the first two items of the PHQ-9 (17).
- Anxiety Generalized Anxiety Disorders (GAD 2) is a very brief and easy-to-perform initial screening tool for generalized anxiety disorder using a Likert scale (0–3) for each criterion. The GAD−2 includes the first two items of the GAD-7. The GAD-2 score ranges from 0 to 6 (18).

7) Participants' perceptions of the impact of the COVID-19 pandemic on lifestyle changes in seven areas: body weight, physical activity, social activities, amount of healthy food, amount of fast and junk food, sleeping hours, and fertility (Supplementary Table 1).

The Likert scale is as follows: 1, very/strongly negatively affected; 2, negatively affected; 3, as is; 4, positively affected; 5, very/strongly positively affected. The participant's perceptions regarding the effect of the COVID-19 pandemic on the changes in their lifestyle ranged from 7 to 35, indicating strongly negative effects and markedly positive effects, respectively. It's a very subjective question.

### Statistical analysis

The data was analyzed using SPSS version 25, with margin of errors 95% and the level of significance was *p* < 0.05. Qualitative data was presented as frequency and percentage, while quantitative data was presented as mean ± SD, median, and range. A fisher exacts, and chi-squared test (*X*^2^) was used to test the association between categorical variables. Moreover, *t*-test, and MannWhitnet U tests were used to test the association between quantitative variables.

### Ethical issues

All participants provided electronic informed written consent after clarification of the goals, data confidentiality, voluntary participation, and withdrawal. The questionnaire contains no sensitive questions, and the data was collected anonymously. We confirm all relevant ethical guidelines have been followed, and any necessary ethics committee approvals have been obtained. The details of the oversight body that provided exemption for the study described are given below:

The study was conducted in accordance with the ethical standards of the 1964 Declaration of Helsinki. Zagazig University Institutional Review Board (ZU-IRP#9288) reviewed and approved this study.

## Results

Of the 1,434 women surveyed, the 180 who were pregnant during the pandemic (not during the study duration) were excluded, as it would have misled the cause of menstrual changes. Of these, 134 got pregnant after being vaccinated 124 (92.5%) had no complications during the pregnancy, 4 (2.9%) aborted the pregnancy, 2 (1.5%) had preterm labor, 2 (1.5%) had fetal death, and 2 (1.5%) had other complications). Furthermore, out of 56 pregnant women who got infected, 46 (82.1%) had no complications and 10 (17.9%) aborted. Therefore, the total number of enrolled participants in this study was 1,254 women of CBA.

### Sociodemographic characteristics

Out of the studied 1,254 women of CBA, the mean (SD) age was 29.6 (8.5) years old, 634 (50%) were married, 1,104 (88.0%) had a University education or above, 1,064 (84.4%) lived in urban areas, 732 (58.4%) were working, and 573 (45.7%) had a BMI of <18 kg/m^2^ (Table 1). The median of GAD-2 and PHQ-2 was two and the mean ± SD was 2.37 ± 1.9 and 2.24 ± 1.8, respectively. The mean ± SD of total lifestyle changes was 20.2 ± 3.9 with a range of 7–35 (Table 2).

**Table 1:** Demographic characteristics and its relationship with the MCs among COVID-19 vaccinated participants.

	**Total sample T** = **1,254** **No (%)**	**Total vaccinated** **T** = **1,044** **No (%)**	**No MCs T** = **626 (59.9) No (%)**	**MCs 418 (38.4) No (%)**	**X2 (p)**
Age (y)					T test= 5.9
Means ± SD	29.6 ± 8.5	30.4 ± 3.9	29.7 ± 9.1	32.9 ± 7.6	(0.008^*^)
**Marital status**					
Widow	4 (0.3)	4 (0.4)	4 (0.5)	0 (0.0)	
Single	634 (50.6)	508 (48.7)	466 (56.2)	168 (40.9)	{0.001^*^}
Married	578 (46.1)	498 (47.7)	324 (40.8)	234 (55.9)	
Divorced	38 (3.0)	34 (3.3)	20 (2.5)	16 (38)	
**Education**					
Preparatory	16 (1.3)	12 (1.1)	6 (1.0)	6 (1.4)	2.18
Secondary	134 (10.7)	114 (10.9)	72 (11.5)	42 (10.5)	(0.59)
University or postgraduate studies	1104 (88.0)	918 (87.9)	548 (87.8)	370 (88.5)	
**Residence**					11.6
Urban	1064 (84.4)	894 (85.6)	518 (82.7)	376 (90.0)	(0.005^*^)
Rural	190 (15.2)	150 (14.4)	108 (17.3)	42 (10.0)	
**Occupation**					0.15
Working	732 (58.4)	624 (59.8)	376 (60.1)	248 (59.3)	(0.93)
Not working	522 (41.6)	420 (40.2)	250 (39.9)	170 (40.7)	
**BMI (kg/m^2^)**					20.2
<18 (underweight)	40 (3.2)	32 (3.1)	22 (3.5)	10 (2.3)	(0.003^*^)
18- <25 (normal weight)	573 (45.7)	461 (44.2)	229 (47.8)	166 (39.4)	
25- <30 (overweight)	390 (31.1)	324 (31.0)	194 (31.0)	122 (39.7)	
30 or more (obese)	251 (20.0)	227 (21.7)	111 (17.7)	118 (28.2)	
**Nationality**					
Saudi	440 (35.1)	436 (41.8)	230 (36.7)	206 (49.3)	47.1
Egyptian	352 (28.1)	302 (28.9)	210 (33.5)	92 (11.3)	(0.0001^*^)
Sudan	68 (5.4)	62 (5.6)	18 (27.3)	26 (6.2)	
Syria	230 (18.3)	130 (12.5)	12 (18.2)	46 (11.0)	
Libya	103 (8.6)	64 (6.1)	6 (9.1)	18 (4.3)	
Others	56 (4.5)	50 (4.8)	2 (3.0)	30 (7.8)	

* *p < 0.05 there was a statistical significant difference; MCs, menstrual Changes; T, total; SD, standard deviation; X^2^ is the symbol of chi square test; t test = student t test; {} p of fisher exact test*.

**Table 2:** Total scores of GAD-2, PHQ-2, and participants ‘perception toward life style changes during the COVID-19 pandemic and its relationship with the MCs among the studied groups participants.

**The total scores of**	**Total sample T** **=** **1,254 Median (Mean** **±SD)**	**Total vaccinated T** **=** **1,044** **Median (Mean** **±SD)**	**No MCs T** **=** **626 (59.9) Median (Mean** **±SD)**	**MCs T** **=** **418 (38.4)** **Median (Mean** **±SD)**	***P-*** **value**
GAD-2	2 (2.37 ± 1.9)	2 (2.2 ± 1.8)	2 (1.9 ± 1.7)	2 (2.6 ± 1.8)	0.003^*^
PHQ-2	2 (2.24 ± 1.8)	2 (2.3 ± 1.9)	2 (2.1 ± 1.9)	2 (2.6 ± 1.8)	0.004^*^
Total Participants ‘perception toward life style changes during the COVID-19 pandemic	19.2 ± 3.9	20.2 ± 3.9	20.6 ± 3.8	18.2 ± 2.9	0.002^*^
**Among COVID-19 cases**					
	**Total sample T = 1,254 median (mean ±SD)**	**Total COVID-19 cases** ** T = 524** ** median (Mean ±SD)**	**No MCs T = 320 (61.1) median (Mean ±SD)**	**MCs** ** T = 204 (38.9%)** ** median (Mean ±SD)**	***P*-value**
GAD-2	2 (2.37 ± 1.9)	2 (2.9 ± 1.9)	2 (2.4 ± 1.9)	2 (3 ± 1.9)	0.01^*^
PHQ-2	2 (2.24 ± 1.8)	2 (2.4 ± 1.8)	2 (2.3 ± 1.8)	2 (2.6 ± 1.9)	0.08
Total Participants ‘perception toward life style changes	19.2 ± 3.9	19.8 ± 3.7 7–30	20.2 ± 3.8 9–30	19 ± 3.0 7–25	0.00^*^

* *P < 0.05. There was a statistically significant difference. GAD-2 is the Generalized Anxiety Disorders score, PHQ-2 is the Patient Health Questionnaire-2 score, MCs are menstrual changes, and T, total; Sd is the standard deviation*.

### COVID-19 vaccination and the determinants of menstrual changes

#### Demographic characteristics

Out of the studied 1,254 women of CBA, 1,044 (83.3%) were vaccinated. the mean age was 30.4 and 3.9 y SD, 508 (48.7%) were single, 918 (87.9%) had a University education or above, 894 (85.6%) lived in urban areas, 624 (59.8%) were working, and 461 (44.2) had a normal BMI kg/m^2^. Vaccinated women of CBA had 418 (38.4) MCs after COVID-19 vaccination. Statistically significant relationships were observed between the reported MCs and the following variables: age, marital status, level of education, nationality, residence, and BMI (Table 1).

In October 2021, the median GAD-2 and PHQ-2 scores among CBA vaccinated women were two, with mean ± SDs of 2.21.8 and 2.3 ± 1.9, respectively. With a range of 7–3. Meanwhile the mean ± SD of total participants' perceptions of lifestyle change was 20.2 ± 3.9. The total assessment score for GAD-2 and PHQ-2, as well as total lifestyle changes, were found to be significantly higher among vaccinated women of CBA with MCs (Table 2).

Out of the studied women of CBA, 1,044 (83.5%) were vaccinated, 659 (63.1%) were vaccinated from <3 months, and 827 (78%) had received two vaccination doses. The most commonly used vaccines in descending order were Pfizer (482, 46.1%), Oxford-AstraZeneca (254, 24.3%), and Sino pharm (92, 8.8%). Moreover, 293 women (80.6%) reported MCs mainly after the second dose. Among the vaccinated women, 482 (46.1) after taking the Pfizer vaccine, 254 (24.3) AstraZeneca, and 92 (8.8) Sino pharm (Table 3).

**Table 3:** The type, and number of COVID-19 vaccination and the relation to the MCs.

	**COVID-19 Vaccination T** = **1,044 No (%)**	**MCs** **T** = **418** **No (% of total vaccinated)**
**Vaccination**		
• AstraZeneca	254 (24.3)	88 (21.1)
• Jonson and Jonson	24 (2.2)	4 (0.9)
• Septotic light	32 (3.1)	8 (1.9)
• Sepotic−7	34 (3.2)	14 (3.3)
• Senopharm	92 (8.8)	26 (6.2)
• Senophak	32 (3.1)	16 (3.8)
• Moderna	10 (0.9)	2 (0.5)
• Pfizer	482 (46.1)	206 (49.3)
• More than one type	84 (8.1)	48 (11.5)
• I don't know	38 (3.6)	
**No of Vaccinated doses**		
• 1^st^ dose	221 (21.2)	81 (19.4)
• 2 doses	823 (78.8)	293 (80.6)

*MCs, menstrual Changes; T, total; Bold refers to the highest or lowest percentages*.

Among the vaccinated women, 184 women (52.3%) who took the Pfizer vaccine and 68 (24.3%) reported MCs Oxford-AstraZeneca and 48 (13.6%) who were vaccinated by more than one type. The main reported side effects were arm heaviness (774, 74.1%), myalgia (550, 52.7%), fever (410, 39.3%), and local redness (250, 23.9); 276 (26.4%) reported no side effects. Moreover, 352 (33.7%) reported changes in the menstrual cycles after vaccination, and 98 (27.8%) reported amenorrhea. The majority of the reported MCs resolved (194, 55.1%) after more than 9 months (Table 4).

**Table 4:** COVID-19 vaccination, and related menstrual changes among the studied cases.

	**COVID-19 vaccination T** = **1,044 (83.5) No (%)**	**SARS-CoV-2 infection T** = **524 (41.8) No (%)**
**Vaccinated/infected since**	**Vaccinated since**	**Infected since**
<3 m	659 (63.1)	100 (19.0)
3 - <6m	294 (28.1)	80 (15.3)
6 - <9 m	62 (5.9)	168 (32.1)
9 - <1 y	29 (2.7)	176 (33.5)
**The required management for infected cases (T = 524)**		
At home		490 (93.5)
At hospital		28 (5.3)
Required ICU		6 (1.1)
**Side effects after vaccinations among COVID-19 vaccinated participants**		
**(T = 1,044)**		
No	270 (25.9)	
Yes	774 (74.1)	
**Main reported side effects among COVID-19 vaccinated participants**		
**(T = 1,044)**		
Local redness	250 (23.9)	
Arm heaviness	774 (74.1)	
Fever	410 (39.3)	
Myalgia	550 (52.7)	
Rhinorrhoea	72 (6.9)	
Headache	142 (13.6)	
**Menstrual changes**	**After vaccination**	**After infection**
MCs	352 (33.7)	98 (18.7)
Amenorrhea	98 (27.8)	26 (4.9)
**Menstrual changes resolved after**	**After vaccination**	**After infection**
1 month	38 (10.8)	18 (18.4)
3 - <6 m	84 (23.9)	6 (6.1)
6 - <9 m	32 (9.1)	4 (4.1)
9 or more	4 (1.1)	0 (0.0)
Still present	194 (55.1)	70 (71.4)

*MCs, menstrual Changes; T, total; m, months; ICU, intensive care unit*.

MCs were observed 48 (57.1%) after more than one type of vaccine, 184 (38.2%) after Pfizer, 12 (37.5%) after Senophak, 10 (29.4%) after Sepotic-7, 8 (25.0%) after Septotic light, 24 (9.4%) after AstraZeneca vaccinations, 4 (16.7%) after J & J vaccinations, 16 (17.4%) after Senopharm, and 2 (20%) after Moderna (Table 4).

**Figure 2:**
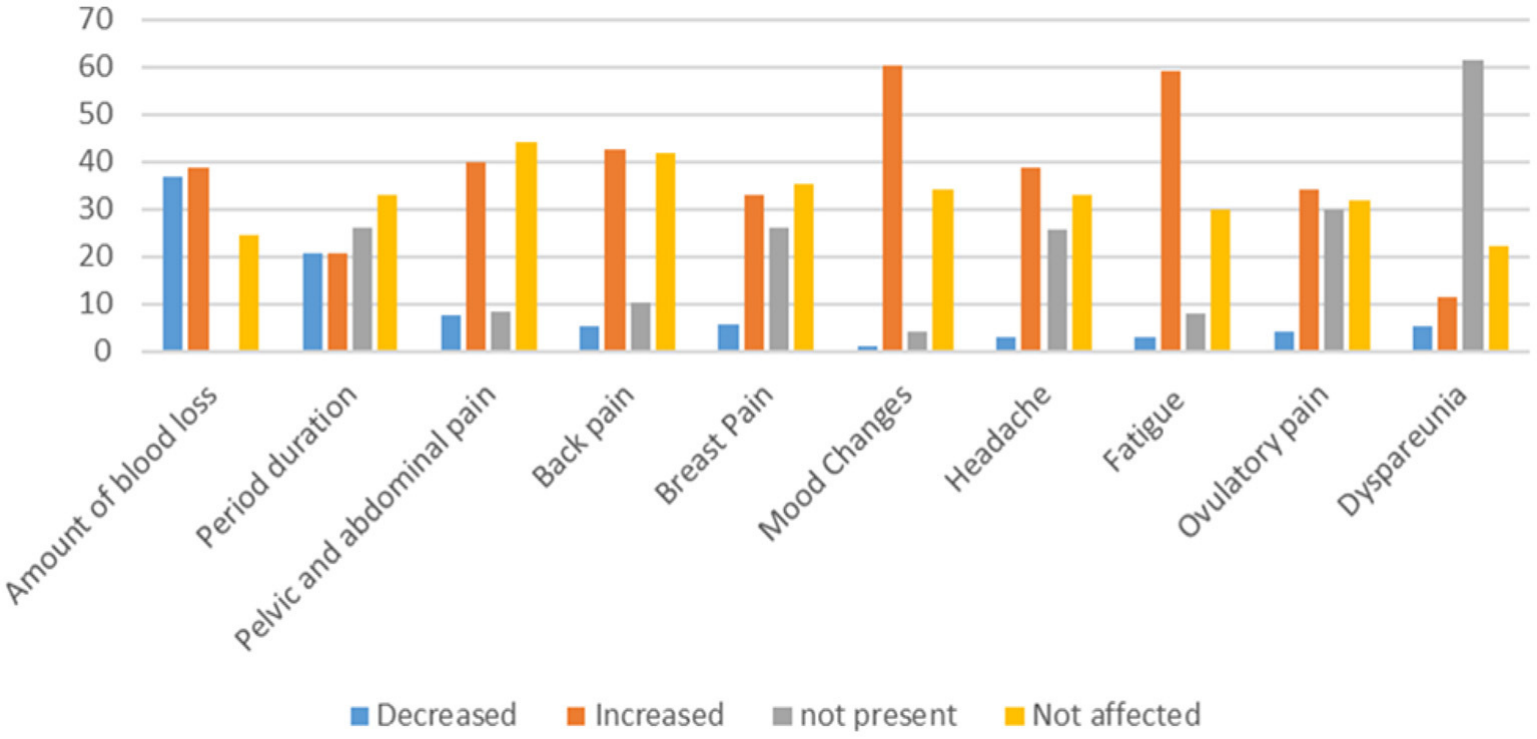
Changes in the menstrual related symptoms among vaccinated women of CBA against SARS-CoV-2 virus.

Regarding the MCs among vaccinated women of CBA, the amount and duration of MCs decreased in 130 (3.9%) and 72 (20.5%), respectively. The mood changes, fatigue, and back pain increased in 212 (60.4%), 208 (59.1%), and 150 (42.6%), respectively (Figure 2).

### SARS-CoV-2 infection and the determinants of menstrual changes

COVID-19 infection was found in 524 (41.8 percent) of the 1,254 women of CBA. The average age was 29.4 years old, with a standard deviation of 8.3 years. 272 (51.0%) were married, 456 (87.0%) had a University education or higher, 444 (84.7%) resided in urban, 296 (65.5%) worked, and 266 (50.8%) had a normal BMI kg/m2. After COVID-19 infection, 98 (18.7%) of the COVID-19-infected women developed MCs, and 106 (20.2%) developed MCs after vaccination. The following characteristics were shown to have statistically significant relationships with reported changes in the menstrual cycle: age, marital status, level of education, nationality, and the BMI (Table 5).

**Table 5:** Demographic data and its relationship with the MCs among COVID-19 cases.

	**Total COVID-19 cases T** = **524 No (%)**	**No MCs** **T** = **320 (61.1)** **No (%)**	**MCs T** = **204 (38.9%) No (%)**	**MCs after vaccine** **T** = **106 (20.2)** **No (%)**	**X2 (** ***p*** **-value)**
**Age (y)**					5.7 (0.03^*^)
Means ± SD (range)	29.4 ± 8.3 (15–50)	28.6 ± 8.2 (15–50)	31.3 ± 8.2 (15–49)	31.3 ± 8.2 (17–49)	
**Marital status**					15.5 (0.02^*^)
Widow	2 (0.4)	2 (0.6)	0 (0.0)	0	
Married	272 (51.0)	186 (58.1)	38 (38.1)	48 (45.3)	
Single	234 (44.7)	124 (38.8)	56 (57.1)	54 (50.9)	
Divorced	16 (3.1)	8 (2.5)	4 (4.1)	4 (3.8)	
**Education**					11.4 (0.02^*^)
Preparatory	6 (1.1)	0 (0.0)	2 (2.0)	4 (3.8)	
Secondary	62 (11.8)	38 (11.1)	10 (10.0)	14 (13.2)	
University or above	456 (87.0)	(88.1)	86 (88.0)	88 (83.0)	
**Residence**					5.50 (0.06)
Urban	444 (84.7)	262 (81.9)	86 (87.8)	96 (90.6)	
Rural	80 (15.3)	58 (18.1)	12 (12.2)	10 (9.3)	
**Occupation**					2.64 (0.27)
Working	296 (65.5)	180 (56.3)	50 (51.0)	66 (62.3)	
Not working	223 (43.5)	140 (43.8)	48 (49.0)	40 (37.7)	
**BMI (kg/m^2^) (20)**					14.3 (0.03^*^)
<18	14 (2.7)	12 (3.8)	0 (0.0)	2 (1.9)	
18 - <25	266 (50.8)	176 (55.0)	44 (4.0)	48 (45.3)	
25 - <30	142 (27.1)	82 (25.6)	30 (30.0)	30 (28.3)	
30 or more	102 (19.5)	50 (15.6)	26 (26.0)	26 (24.5)	
Nationality					57.9 (0.00^*^)
Saudi	122 (23.2)	56 (17.5)	26 (26.5)	40 (37.7)	
Egyptian	168 (32.1)	104 (32.5)	30 (30.6)	34 (32.1)	
Sudan	22 (4.2)	18 (5.6)	2 (2.0)	2 (1.9)	
Syrian	128 (24.4)	98 (30.9)	20 (20.4)	10 (9.4)	
Libyan	58 (11.1)	36 (11.3)	22 (10.8)	6 (5.7)	
Others	26 (5.0)	8 (2.5)	16 (7.8)	14 (13.2)	

* *p < 0.05 there was a statistical significant difference. BMI, Body mass index; T, total; SD, standard Deviation; MCs, menstrual Changes; X^2^ is the symbol of chi square test. Values in bold indicates the highest or lowest percentages*.

The median of GAD-2 and PHQ-2 among vaccinated women of CBA was two and the mean ± SD was 2.9 ± 1.9 and 2.4 ± 1.8, respectively. The mean ± SD of total lifestyle changes was 19.8 ± 3.7 with a range of 7–30. The total assessment scores for GAD-2 and PHQ-2 (significantly higher among infected females with menstrual disturbance after being vaccinated 2 (3 ± 1.9) and 2 (2.6 ± 1.7), respectively), and total lifestyle changes (the lowest among women reporting changes after being vaccinated) (Table 2).

Out of the studied women, 524 (41.8%) were infected with SARS-CoV-2, 180 (34.3%) were infected from <6 m, 494 (94.2%) were treated at home, 98 (18.7%) reported MCs after infection, and 26 (4.9%) reported amenorrhea. The reported changes in the menstrual cycle resolved after 1 month in 18 women (18.4%) and are still present in 70 (71.4%) (Table 4).

**Figure 3:**
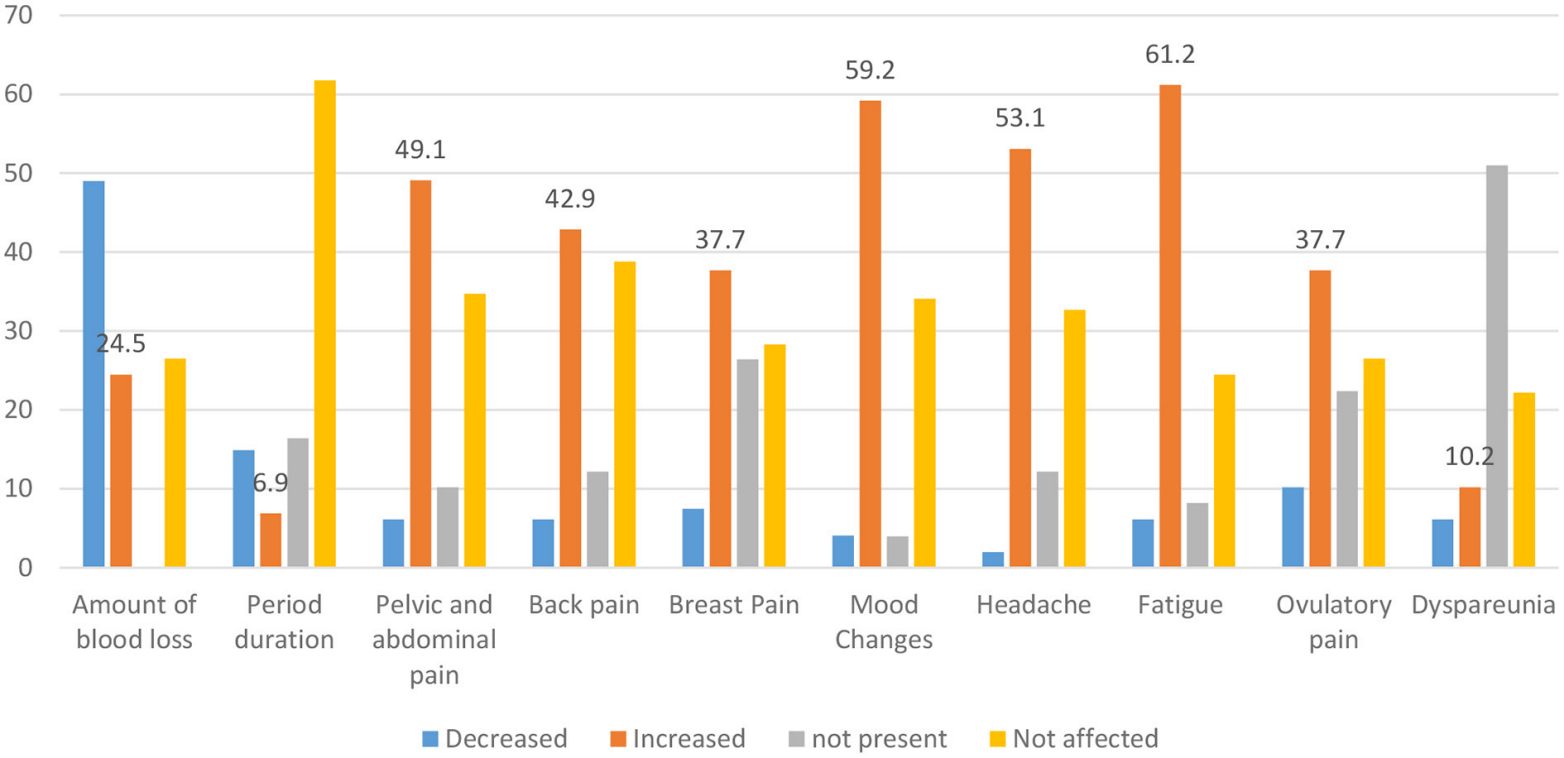
Changes in the menstrual related symptoms among COVID-19 cases.

Regarding the changes in the menstrual cycles among infected women of CBA, the amount of menstruation decreased in 48 women (49.0%), whereas fatigue, mood changes, headache, and pelvic and abdominal pain increased in 60 women (61.2%), 58 (59.2%), 52 (53.1%), and 48 (49.1%), respectively. The menstrual cycle duration was the highest unaffected parameter after COVID-19 disease (Figure 3).

## Discussion

Since the start of the COVID-19 pandemic, there has been a growing body of evidence on social media and blogs that women have experienced MCs such as altered menstrual duration, frequency, regularity, and volume (heavier bleeding and clotting), increased dysmenorrhea, and worsened premenstrual syndrome (PMS). Recent anecdotal reports about the MCs following COVID-19 vaccination have fuelled vaccine hesitancy or refusal. A proper and factual scientific investigation of these phenomena is critical for the public health (12).

### MCs after COVID-19 vaccination

As shown in Table 3, the most commonly used vaccines in descending order were Pfizer (482, 46.1%), AstraZeneca (254, 24.3%), and Sino pharm (92, 8.8%). The Pfizer-BioNTech vaccine was the first COVID-19 vaccine that received the Food and Drug Administration (FDA) approval for Emergency Use Authorization (EUA) in December 2020 after it effectively prevented the disease symptom and the first that received the full FDA approval in August 2021 for people of 16 years and older (21).

Menstrual irregularities were found in 35.8% of the COVID-19-vaccinated women, 26.7% of women vaccinated with a single dose, and 23.5% of those vaccinated with two doses of the vaccine (Table 3). This can be explained by the NIH reports that each dosage of the COVID-19 vaccination resulted in a nearly one-day increase in cycle length. The first dosage of a two-dose vaccine was linked to a 0.71-day increase in cycle length, while the second dose was linked to a 0.91-day increase^3^.

About 26% of the vaccinated females reported no side effects, and the main reported side effects (Table 4) were arm heaviness (774, 74.1%), myalgia (550, 52.7%), fever (410, 39.3%), and local redness (250, 23.9%). In agreement with the CDC report (22), the main temporary side effects were an unsightly arm rash or lymph node swelling called the “COVID arm” and because these reactions are normal, medical experts wanted to get the word out to avoid alarming those who experience such symptoms. These symptoms are normal, may affect the daily activities, and resolve within a few days as the females' body build protection.

The majority of vaccinated women with MCs reported persistent irregularities after 9 months (194, 55.1%) (Table 4). The menstrual cycle of the studied cases does not become regular within a reasonable time due to the factors discussed above. On the other hand, (23) reported that the length of menstrual cycles after vaccination decreased in consecutive cycles, indicating that the effects are most likely transient (23).

MCs were observed in 48 (57.1%) after receiving more than one type of vaccine, 184 (38.2%) after receiving Pfizer, 12 (37.5%) after receiving Senophak, 10 (29.4%) after receiving Sepotic-7, and 8 (25.0%) after receiving Septotic Light (Table 4). This is explained by the fact that MCs have been recorded following both mRNA and adenovirus vectored COVID-19 vaccines, implying that, if there is a link, it is most likely due to the immunological response to immunization rather than a specific vaccine component. Research into a suspected link between COVID-19 vaccinations and MCs could also aid in the discovery of the mechanism (7, 13, 24).

We reported that more than 35% of the studied vaccinated women reported changes in the duration of their menstrual cycles (Figure 1). The length of a menstrual period varies a lot, and specialists say that variations of up to 8 days are common. The prolonged menstrual periods that occurred following immunization became shorter in subsequent cycles (23). This is inconsistent with what was reported by MHRA; analysis of Yellow Card reports does not support a link between changes in menstrual periods and COVID-19 vaccines. However, the data collection method of the Yellow Card system makes it difficult to draw firm conclusions (6). An appropriate approach for a better comparison between the rates of MCs among vaccinated vs. unvaccinated populations was offered by the US National Institutes of Health, which provided around $1.67 million to encourage this research (25).

NB: Despite the earlier-mentioned 180 excluded women who due to pregnancies, it should be noted that the COVID-19 vaccine builds antibodies that reduce the risk of COVID-19 infection and might protect babies (26–28), and there are early reassuring data on the safety of (Pfizer and Modern) mRNA vaccine. Still, in agreement with our results, the CDC recommends following up with vaccinated pregnant females during all trimesters for better understanding of the effects of the vaccine on pregnancy and babies through the enrolment in v-safe (pregnancy registry through the CDC's smartphone-based tool) to provide individualized health check-ins after being vaccinated (29).

### MCs after COVID-19 diseases

Out of the studied females, 524 (41.8%) gave a history of SARS-CoV-2 Infection. MCs were reported in 98 women (18.7%) after infection; 26 (4.9%) reported amenorrhea (Table 4). Regarding the mood of menstrual irregularities, in 98 women, hypomenorrhea (decreased amount of period) was the most common form (48, 49.0%) and amenorrhea was less frequent (26, 26.5%) (Figure 2). This finding is consistent with a Chinese study conducted by Li et al. (13), who have found that out of the 237 studied infected women, 59 (25%) reported MCs: 47 (20%) with decreased menstrual volume and only 12 (5%) with increased volume.

Furthermore, Davis et al. (30), in a global multinational survey including 2,961 women [76% from the United States of America (USA) and United Kingdom (UK)], have reported abnormally irregular menstrual cycles (26%), for example, abnormally heavy periods or clotting in 592 women (20%). Moreover, another interesting observation in their study is that 89 women (3%) experienced early menopause among women in their 40s (30).

Although the exact mechanism by which menstrual irregularities occur after SARS-CoV-2 Infection is unclear, it can be explained by the effect of COVID-19 illness on changing the hypothalamic-pituitary-ovarian (HPO) axis and endometrial function. COVID-19 directly affects the female reproductive system through Angiotensin-Converting Enzymes II (ACE2) receptors, which are found on ovarian and endometrial tissues (31). The HPO axis (which regulates the menstrual cycle) is disturbed by an energy deficiency and stress. COVID-19 disease leads to severe energy deficiency, which disrupts the luteal phase and may cause anovulation. This leads to oligo-hypo menorrhea (infrequent scanty periods). The reasons may range from tapering workload, anxiety and stress, to disease-linked inflammation and immune alterations (2). The HPO axis controls the menstrual cycles by negative and positive feedback mechanisms between the hypothalamus, anterior pituitary, and ovaries (32, 33).

Post-COVID-19, amenorrhea was reported by 26 (4.9%) women (Table 4). Although the exact mechanism is still unknown, many hypotheses have been proposed, most of which are related to hypothalamic-pituitary causes, such as the reported high pandemic psychological effect on anxiety levels and the reported changes in 388 (74.0%) negatively affected body weight in dietary habits, which may result in dysthymic GnRH releases and transient menstrual changes (34). As long as we excluded pregnant women and those who had any cause for MCs.

We reported that the MCs resolved after 1 month in only 18 women (18.4% of infected women with MCs) and are still present in 70 (71.4%). The MCs of these patients might be a consequence of transient sex hormone changes caused by suppression of ovarian function that quickly resumes after recovery (13) (Table 4).

Mood Determinates of MC after SARS-CoV-2 Infection; The total GAD-2 and PHQ-2 scores were significantly higher among infected women with menstrual disturbances after being vaccinated (Table 2). In different countries, anxiety about COVID-19 was reported, as infected persons can be subjected to social discrimination and stigma. Moreover, COVID-19 could have negative effects on a person's mental, social, and physical health due to social rejection, the prevalence of all types of violence against women and their children was increasingly reported (35, 36), and access to many services, for example, educational and health services, was limited (37). In addition, changes in lifestyle among infected cases were augmented in terms of the level of stress.

The perception of women of CBA regarding the Lifestyle changes as a determinates of MC after SARS-CoV-2 Infection. The most negatively affected lifestyle domains among COVID-19-affected females in CBPs were physical activity (492, 38.1 percent) and body weight (31.0 percent) as in Supplementary Materials. Indeed, during the pandemic, men and women showed a decrease in physical activity levels (38). Step counts decreased worldwide in the period after COVID-19 was declared a global pandemic. Differences were observed between regions, which are most likely due to regional variation in COVID-19 timing, regional enforcement, and behavior change (39). For example, in Brazil (June 2020), physical inactivity was 46% among females (38).

The COVID-19 pandemic had a negative impact on 37.1 percent of women of CBA s' social activities. This is because it was at the peak of COVID-19 restrictions. Face-to-face interactions were often limited to core network members, such as spouses, family members, or possibly live-in roommates; some “weak” relationships were lost, and interactions were restricted to those closest to them. Given that peripheral, weaker social relationships provide a diversity of resources, opinions, and support, COVID-19 most likely led to networks that were smaller and more homogeneous (40). This should affect the females' moods and raise their stress, anxiety, and despair levels, especially among previously infected females (41).

Most of the studied women of CBA during the fourth wave of the COVID-19 pandemic (1092, 87.1%) perceived that the SARS-Con-2 infection and or COVID-19 vaccination did not affect their fertility (Supplementary Materials). This is because there were no significant reported changes in the concentration of the sex hormone or even the ovarian reserve among COVID-19-infected women of CBA (13). This result is consistent with that of the study by Male et al., who reported that there was no evidence that COVID-19 vaccination has an adverse effect on fertility. Unintended pregnancies occurred in clinical trials at comparable rates in vaccinated and unvaccinated groups (8). In assisted reproduction clinics, fertility measures and pregnancy rates are similar in vaccinated and unvaccinated patients (42–44).

Due to all the above-mentioned factors, including violence, stressors, lifestyle changes, poor access to health services, as estimated by Marie Stopes International, the COVID-19 pandemic prevented this year up to 9.5 million girls and women around the world from accessing health services (45), which may have played a role in the reported abortion cases (10, 17.9%).

### Strength

This study responds to the NIH call to researchers on September 30, 2021 to investigate the MCs after the COVID-19 vaccine. A major strength of this study is the large number of women surveyed from across multiple Arabic countries after being validated. Also the survey was conducted anonymously online, limiting the potential effects of social desirability and cultural bias. This study addresses the post-COVID-19 diseases or vaccination sequelae and studies seven types of vaccines. Our study included both hospitalized and non-hospitalized patients, symptomatic and asymptomatic COVID-19 cases. We were able to document the scope of the issue and identify a number of predictors that led to MCs. Our study also used multiple tools to identify the consequences of the COVID-19 disease or vaccine Patients from an earlier wave of COVID-19 disease were included in our research. We were also able to measure parameters related to healthcare resource utilization, such as revisit rates to healthcare facilities and readmissions. However, our study has a number of limitations.

### Limitations

The main reported limitations were as follows: Our study has all of the limitations associated with observational cross-sectional retrospective studies, such as bias, confounding, and lack of control group. Second, because of recall and availability bias, the study used an online self-administered survey, which leads to selection sampling bias as the majority of participants were highly educated, and may affect the quality and generalizability of the collected data. The lack of knowledge of SARS-CoV-2 infection or COVID-19 vaccination effects during this time period may have influenced the reporting of relevant data. When evaluating pre-COVID-19 infection or vaccine baseline menstrual changes, it is impossible to distinguish pre-existing abnormalities from those attributable to SARS-CoV-2 infection and/or COVID-19 vaccination. Despite these limitations, our findings are backed up by findings from a number of international investigations. In addition to that, approximately 87.6% of the enrolled women were highly educated in University or postgraduate studies. Arabic women's cultural, social, health, and religious beliefs play a big role in pushing all women, even those single or illiterate, to track and keen on the regularity of their menstrual cycle.

Some participants aren't likely to report these MCs unless they are asked directly after getting the COVID-19 vaccine or getting infected. Women who couldn't read or write and people who didn't use the Internet were also left out.

The size of the samples from different countries varies dramatically due to two main factors (1) The sample size was estimated based on many determinants as illustrated in the methodology, including that since the overall population of Egypt was more than 110 million, Saudi Arabia was about 34 million, and Syria was about 17 million. The total number of CBA women in each nation (which varies greatly). Because COVID-19 testing was easy to get and free, the number of infected people in Saudi Arabia rose to 549,222, while it was only 41,093 in Sudan. The number of people who were vaccinated was influenced by vaccine availability dates (vaccination in Syria began only a month before the research) and vaccination coverage rates, which were highest in Saudi Arabia. (2) Aside from the political turmoil in Syria and Sudan, which affects how well and how often people use the Internet, the response rate from Syria and Sudan was very low because the sample was gathered through an online questionnaire link. Finally, another weakness of our study was convenience sampling and not all Arabic countries participated, which may limit the generalizability.

## Conclusion

MCs among Women of CBA after SARS-CoV-2 infection and /or COVID-19 vaccination are a prevalent and complex problem. Nearly one-quarter of the COVID-19 women cases and one-third vaccinated women reported MCs after infection and or vaccination. Moreover, age, marital status, level of education, nationality, BMI, and the total assessment scores for GAD-2 and PHQ-2 significantly affect these MCs.

## Recommendations

1) *At the Research Level*. (a) Further detailed prospective investigations and researches are essential to provide accurate information using routinely collected clinical data to reduce selection and recall bias. (b) Information about menstrual cycles and abnormal vaginal bleeding should be actively solicited in future clinical trials, including trials of COVID-19 vaccines. (c) Cohort studies should be conducted with controlling the confounding factors based on the outcome of interest and exposure, for example, the use of hormonal contraceptives. (d) Ideal setting clinical trials should be performed to differentiate between MCs caused by interventions from those that occur anyway. (e) Further studies involving MCs after vaccination with randomization.
2) *At the Vaccination Level*. (a) The level of awareness about the importance of vaccines should be increased; if there are any inquires in English or Spanish, Call 1-866-626-6847 and chat live or send an email to MotherToBaby. (b) An accessible, effective, national ongoing safety assessment should be established to adequately assess vaccination safety and improve the outcome of immunization programs. Although existing comprehensive systems to monitor vaccine safety are in place, they are being enhanced for the rollout of the COVID-19 vaccine program. It is particularly important to identify rare adverse events that are causally related to vaccine administration and assess their incidence and risk factors to inform potential vaccine contraindications.
3) *For Vaccinated Pregnant Women*. We encourage the voluntary use of the pregnancy registry v-safe at the time of vaccination or after vaccination to gather information on the health of pregnant women. The registry staff contracted by the CDC might contact the participants.
4) *At the Policy Level*. The meaningful participation of women and girls must be ensured for proper decision-making and effective implementation of any national or international programs.
5) *At the Principle Level*. We encourage respecting the principle of “we are all in this together” as in the United Nations brief on COVID-19 and human rights should be considered (45).
6) *At the Preparedness Level*. For any national and international global strategic plans, the gender analysis must be strongly grounded.
7) *At the Level of Services Access*. As a part of any pandemic and crisis, the impact of violence on women and their children should be critically mitigated by ensuring access to essential services for survivors of violence.

## Data availability statement

The original contributions presented in the study are included in the article/supplementary material, further inquiries can be directed to the corresponding authors.

## Ethics statement

The studies involving human participants were reviewed and approved by Zagazig University Institutional Review Board (ZU-IRP#9288). The patients/participants provided their written informed consent to participate in this study.

## Author contributions

Conceptualization: AAm, SA, and MR. Data curation and investigation: KA, BA, DY, NA, AAl, and MR. Formal analysis: SA. Methodology and supervision: AAm and SA. Project administration: KA and BA. Software: JS. Validation: SA and MR. Visualization: JS and MR. Writing—original draft preparation: AAm, SA, and EA-E. All authors: writing—review and editing, contributed to the article, and approved the submitted version.

## Conflict of interest

The authors declare that the research was conducted in the absence of any commercial or financial relationships that could be construed as a potential conflict of interest.

## Publisher's note

All claims expressed in this article are solely those of the authors and do not necessarily represent those of their affiliated organizations, or those of the publisher, the editors and the reviewers. Any product that may be evaluated in this article, or claim that may be made by its manufacturer, is not guaranteed or endorsed by the publisher.

## Footnotes

^1^Available online at: https://www.health.gov.au/news/health-alerts/novel-coronavirus-2019-ncov-health-alert/coronavirus-covid-19-case-numbers-and-statistics.

^2^Available online at: https://coronavirus.jhu.edu/map.html.

^3^Available online at: https://www.nih.gov/news-events/nih-research-matters/covid-19-vaccines-linked-small-increase-menstrual-cycle-length.

## Abbreviations

NICHD: National Institute of Child Health and Human Development
COVID-19: Coronavirus disease 2019
FDA: Food and Drug Administration
EUA: Emergency Use Authorization
WHO: The World Health Organization
MCs: Menstrual changes
NICHD: Eunice Kennedy Shriver National Institute of Child Health and Human Development
CBP: Child bearing Periods
HPO: Hypothalamic-pituitary-ovarian
PHQ-2: Patient Health Questionnaire-2
GAD −2: Generalized Anxiety Disorders
CDC: Center of Disease Control
PHO: Pituitary Hypothalamic-ovarian
MHRA: Medicines and Healthcare Products Regulatory Agency
ACE2: Angiotensin-Converting Enzymes II.

## References

[1] Huang C Wang Y Li X Ren L Zhao J Hu Y. Clinical features of patients infected with 2019 novel coronavirus in Wuhan, China. Lancet. (2020) 395:497–506. 10.1016/S0140-6736(20)30183-5 31986264 PMC7159299

[2] Demir O Sal H Comba C. Triangle of COVID, anxiety and menstrual cycle. J Obstet Gynaecol. (2021) 41:1257–61. 10.1080/01443615.2021.1907562 33955327

[3] Yuksel B Ozgor F. Effect of the COVID-19 pandemic on female sexual behavior. Int J Gynaecol Obstet. (2020) 150:98–102. 10.1002/ijgo.13193 32392400 PMC9087619

[4] Takmaz T Gundogmus I Okten SB Gunduz A. The impact of COVID-19-related mental health issues on menstrual cycle characteristics of female healthcare providers. J Obstet Gynaecol Res. (2021) 47:3241–9. 10.1111/jog.14900 34137123 PMC8447072

[5] World Health Organization. Draft Landscape of COVID-19 Candidate Vaccines. Available online at: https://www.who.int/publications/m/item/draft-landscape-of-covid-19-candidate-vaccines (accessed October 20, 2020).

[6] Medicines Healthcare Products Regulatory Agency. Coronavirus Vaccine—Weekly Summary of Yellow Card Reporting. (2021). Available online at: https://www.gov.uk/government/publications/coronavirus-covid-19-vaccine-adverse-reactions/coronavirus-vaccine-summary-of-yellow-card-reporting#annex-1-vaccine-analysis-print

[7] Karagiannis A Harsoulis F. Gonadal dysfunction in systemic diseases. Eur J Endocrinol. (2005) 152:501–13. 10.1530/eje.1.01886 15817904

[8] Male V. Are COVID-19 vaccines safe in pregnancy? Nat Rev Immunol. (2021) 21:200–1. 10.1038/s41577-021-00525-y 33658707 PMC7927763

[9] Speed B. Young Women are the Unlikely New Face of Covid-19 Vaccine Resistance. i News (2021). Available online at: https://inews.co.uk/news/health/coronavirus-latest-experts-debunk-vaccine-fertility-myths-women-819783

[10] Medicines Healthcare Products Regulatory Agency. COVID-19 Vaccines: Updates for August 2021. Available online at: https://www.gov.uk/drug-safety-update/covid-19-vaccines-updates-for-august-2021

[11] Royal College of Obstetricians Gynecologists. RCOG Responds to Reports That COVID-19 Vaccine Affects Periods. (2021). Available online at: https://www.rcog.org.uk/en/news/rcog-responds-to-reports-that-covid-19-vaccine-affects-periods/

[12] Sharp G Fraser A Sawyer G Kountourides G Easey K Ford G. The COVID-19 pandemic and the menstrual cycle: research gaps and opportunities. Int J Epidemiol. (2022) 51:691–700. 10.1093/ije/dyab239 34865021 PMC8690231

[13] Li K Chen G Hou H Liao Q Chen J Bai H. Analysis of sex hormones and menstruation in COVID-19 women of child-bearing age. Reprod Biomed Online. (2021) 42:260–7. 10.1016/j.rbmo.2020.09.020 33288478 PMC7522626

[14] Food agricultural organizations of the United States. Gendered Impacts of COVID-19 and Equitable Policy Responses in Agriculture, Food Security and Nutrition. Available online at: https://reliefweb.int/sites/reliefweb.int/files/resources/CA9198EN.pdf (accessed May 5, 2020).

[15] Gender and COVID-19. WHO-2019-nCoV-Advocacy_brief-Gender-2020.1-eng.pdf, Advocacy brief (2020).

[16] WHO. Women of Reproductive Age (15-49 years) Population. Available online at: https://www.who.int/data/gho/indicator-metadata-registry/imr-details/women-of-reproductive-age-(15-49-years)-population-(thousands) (accessed November 15, 2021).

[17] Patient Health Questionnaire-2 (PHQ-2). Mental Disorders Screening - National HIV Curriculum. Available online at: https://www.hiv.uw.edu/page/mental-health-screening/phq-2

[18] Kroenke K Spitzer RL Williams JB Monahan PO Löwe B. Anxiety disorders in primary care: prevalence, impairment, comorbidity, and detection. Ann Intern Med. (2007) 146:317–25. 10.7326/0003-4819-146-5-200703060-00004 17339617

[19] Bain J Bragg S Ramsetty A Bradford S. Endocrine conditions in older adults: menopause. FP Essent. (2018) 474:20–7. 30427649

[20] CDC about Adult BMI. Healthy Weight, Nutrition, and Physical Activity. Available online at: https://www.cdc.gov/healthyweight/assessing/bmi/adult_bmi/index.html (accessed November 15, 2021).

[21] Emergency Use Authorization (EUA) of the Pfizer-BioNTech COVID-19 Vaccine to Prevent Coronavirus. Fact Sheet for Healthcare Providers Administering Vaccine. Available online at: https://www.fda.gov/media/144413/download (accessed October 21, 2021).

[22] Possible Side Effects After Getting a COVID-19 Vaccine (CDC) (2021). Yale Medicine Vaccine Content Center > News > Yale Medicine.

[23] Edelman A Boniface ER Benhar E Han L Matteson KA Favaro C. Obstet gynecol. association between menstrual cycle length and coronavirus disease 2019 (COVID-19) vaccination: a U.S. cohort. Obstet Gynecol. (2022) 139:481–9. 10.1097/AOG.0000000000004695 34991109 PMC8936155

[24] Monin L Whettlock EM Male V. Immune responses in the human female reproductive tract. Immunology. (2020) 160:106–15. 10.1111/imm.13136 31630394 PMC7218661

[25] Eunice Kennedy Shriver National Institute of Child Health Human Development. Item of Interest: NIH Funds Studies to Assess Potential Effects of COVID-19 Vaccination on Menstruation. (2021). Available online at: https://www.nichd.nih.gov/newsroom/news/083021-COVID-19-vaccination-menstruation

[26] Goldshtein I Nevo D Steinberg DM Rotem RS Gorfine M Chodick G. Association between BNT162b2 vaccination and incidence of SARS-CoV-2 infection in pregnant women. JAMA. (2021) 326:728–35. 10.1001/jama.2021.11035 34251417 PMC8276131

[27] Dagan N Barda N Biron-Shental T Makov-Assif M Key C Kohane IS. Effectiveness of the BNT162b2 mRNA COVID-19 vaccine in pregnancy. Nat Med. (2021) 27:1693–5. 10.1038/s41591-021-01490-8 34493859

[28] Gray KJ Bordt EA Atyeo C Gray KJ Bordt EA Atyeo C. Coronavirus disease 2019 vaccine response in pregnant and lactating women: a cohort study. Am J Obstet Gynecol. (2021) 225:303.e1-17. 10.1101/2021.03.07.21253094 33775692 PMC7997025

[29] COVID-19 Vaccines While Pregnant or Breastfeeding (cdc.gov) (2021).

[30] Davis HE Assaf GS McCorkell L Wei H Low RJ Re'em Y. Characterizing long COVID in an international cohort: 7 months of symptoms and their impact. EClinicalMedicine. (2021) 38:101019. 10.1016/j.eclinm.2021.101019 34308300 PMC8280690

[31] Kong S Yan Z Yuan P Liu X Chen Y Yang M. Comprehensive evaluation of ACE2 expression in female ovary by single-cell RNA-seq analysis. Developmental Biology (2021). Available online at: http://biorxiv.org/lookup/doi/10.1101/2021.02.23.432460 (accessed May 21, 2021).

[32] Mountjoy M Sundgot-Borgen J Burke LM Ackerman KE Blauwet C Constantini N. IOC consensus statement on relative energy deficiency in sport (RED-S): 2018 update. Int J Sport Nutr Exerc Metab. (2018) 28:316–31. 10.1123/ijsnem.2018-0136 29771168

[33] Edwards C Thornton J. Athlete Mental Health and Mental Illness in the Era of COVID-19: Shifting Focus With a New Reality BJSM Blog. Available online at: https://blogs.bmj.com/bjsm/2020/03/25/athlete-mental-health-and-mental-illness-in-the-era-of-COVID-19-shifting-focus-a-new-reality/ (accessed March 25, 2020).

[34] Sharp GC Fraser A Sawyer G Kountourides G Easey KE Ford G. The COVID-19 pandemic and the menstrual cycle: research gaps and opportunities. Int J Epidemiol. (2021) 51:691–700. 10.31219/osf.io/fxygt 34865021 PMC8690231

[35] COVID-19 and Violence Against Women: What the Health Sector/System Can Do. Geneva: World Health Organization (2020). Available online at: https://apps.who.int/iris/handle/10665/331699 (accessed April 28, 2020).

[36] Reducing stigma [website]. Atlanta, GA: Centers for Disease Control Prevention (2020). Available online at: https://www.cdc.gov/coronavirus/2019-ncov/dailylife-coping/reducingstigma.html?CDC_AA_refVal=https%3A%2F%2Fw ww.cdc.gov%2Fcoronavirus%2F2019- ncov%2Fsymptoms-testing%2Freducing-stigma.html (accessed April 28, 2020).

[37] Buller AM Peterman A Ranganathan M Bleile A Hidrobo M Heise L. A mixed-method review of cash transfers and intimate partner violence in low- and middle-income countries. World Bank Res Observ. (2018) 33:218–58. 10.1093/wbro/lky002

[38] Puccinelli PJ da Costa TS Seffrin A de Lira CAB Vancini RL Nikolaidis PT. Reduced level of physical activity during COVID-19 pandemic is associated with depression and anxiety levels: an internet-based survey. BMC Public Health. (2021) 21:425. 10.1186/s12889-021-10470-z 33648487 PMC7919983

[39] Tison GH Avram R Kuhar P Abreau S Marcus GM Pletcher MJ. Worldwide effect of COVID-19 on physical activity: a descriptive study. Ann Intern Med. (2020) 173:767–70. 10.7326/M20-2665 32598162 PMC7384265

[40] Long E Patterson S Maxwell K Blake C Pérez RB Lewis R. COVID-19 pandemic and its impact on social relationships and health. J Epidemiol Community Health. (2022) 76:128–32. 10.1136/jech-2021-216690 34413184 PMC8380476

[41] Attia MA Mohamed FA Allah MAEA Khorkhash MK. Prevalence, symptoms, determinants and coping mechanisms among University students during COVID-19 Pandemic 2021. Clin Psychiatr Neurosci. (2022).

[42] Morris RS. SARS-CoV-2 spike protein seropositivity from vaccination or infection does not cause sterility. F S Rep. (2021) 2:253–5. 10.1016/j.xfre.2021.05.010 34095871 PMC8169568

[43] Orvieto R Noach-Hirsh M Segev-Zahav A Haas J Nahum R Aizer A. Does mRNA SARS-CoV-2 vaccine influence patients' performance during IVF-ET cycle? Reprod Biol Endocrinol. (2021) 19:69. 10.1186/s12958-021-00757-6 33985514 PMC8116639

[44] Bentov Y Beharier O Moav-Zafrir A Kabessa M Godin M Greenfield CS. Ovarian follicular function is not altered by SARS-CoV-2 infection or BNT162b2 mRNA COVID-19 vaccination. Hum Reprod. (2021) 36:2506–513. 10.1093/humrep/deab182 34364311 PMC8385874

[45] The indirect impact of COVID-19 on women Lockdown Measures and School Closures Affect Girls and Women Differently Across the World and May Have Long-Term Negative Consequences. Talha Burki Reports (2020). Available online at: www.thelancet.com/infection

