# Supplementary material for: Menstrual changes after COVID-19 vaccination and/or SARS-CoV-2 infection and their demographic, mood, and lifestyle determinants in Arab women of childbearing age, 2021
Source: Front Reprod Health. 2022 Jul 22;4:927211. doi: 10.3389/frph.2022.927211 (PMC9580647; doi:10.3389/frph.2022.927211)
Supplement: Supplementary file 1 [file Table_1.docx]

**Supplementary Materials**

**Supplementary Table 1;** Participants' perceptions regarding the effect of a pandemic on the following lifestyle domains during October 2021

| Participants' perceptions regarding the effect of the pandemic on the following lifestyle domains | Very/strongly negatively affected No (%) | Negatively  No (%) | As it is  No (%) | Positively  No (%) | very/strongly positively affected  No (%) | Total score  Means±SD  Range |
| --- | --- | --- | --- | --- | --- | --- |
| Body weight | **80(6.4)** | **308(24.6)** | 626(49.9) | 96(7.7) | 144(11.5) | 20.2+_3.9  7-35 |
| Physical activity | **92(7.3)** | **400(31.9)** | 556(44.3) | 36(2.9) | 170(13.6) |  |
| Social activities | **120(6.9)** | **368(29.3)** | 492(39.8) | 66(5.3) | 208(16.6) |  |
| Amount of Healthy food | 62(4.9) | 264(21.1) | 594(47.4) | **62(4.8)** | **264(21.1)** |  |
| FF and JF consumption | 84(6.7) | 270(21.5) | 628(50.1) | **88(7.0)** | **184(14.7)** |  |
| Sleeping hours | 100(8.0) | 290(23.1) | **642(51.2)** | 54(4.3) | 168(13.4) |  |
| The perception that the pandemic affects fertility | 26(2.1) | 66(5.3) | **1092(87.1)** | 38(3.0) | 32(2.6) |  |

Fast and junk food (FF and JF) Bold refers to the highest or lowest percentages

Participants' perceptions regarding the effect of a pandemic on the following lifestyle domains during October 2021 among women of CBA; physical activity (492, 38.1%), social activities (37.1%), and body weight (31.0%) were the main negatively affected domains, whereas the amount of healthy food intake (25.9%), FF, and JF (21.7%) were the most positively affected domains. On the other hand, their perceptions that the COVID-19 pandemic affects fertility (1092, 87.1%) and sleeping hours (642, 51.2%) were the most unaffected domains **(Table 1) in the supplementary materials**
